# Perineural Injection Therapy for Hemiplegic Shoulder Pain: A Novel Management Approach

**DOI:** 10.7759/cureus.79332

**Published:** 2025-02-19

**Authors:** Jern Tung Choong, Anwar Suhaimi, Daniel Chiung-Jui Su, King Hei Stanley Lam

**Affiliations:** 1 Department of Rehabilitation Medicine, Cheras Rehabilitation Hospital, Kuala Lumpur, MYS; 2 Department of Rehabilitation Medicine, Universiti Malaya Medical Centre, Kuala Lumpur, MYS; 3 Department of Rehabilitation Medicine, Universiti Malaya, Kuala Lumpur, MYS; 4 Department of Physical Medicine and Rehabilitation, Chi Mei Medical Center, Tainan, TWN; 5 Faculty of Medicine, The University of Hong Kong, Hong Kong, HKG; 6 Board of Clinical Research, The Hong Kong Institute of Musculoskeletal Medicine, Kowloon, HKG; 7 Faculty of Medicine, The Chinese University of Hong Kong, New Territories, HKG

**Keywords:** 5% dextrose, hemiplegic shoulder pain, landmark guided, minimal clinically important difference, pain management, perineural injection therapy, post-stroke rehabilitation, upper limb rehabilitation

## Abstract

Hemiplegic shoulder pain (HSP) significantly interferes with upper limb rehabilitation and reduces the function and quality of life in stroke patients. Perineural injection therapy (PIT) offers a regenerative approach by targeting potential pain-generating nerves utilizing dextrose. The effectiveness of PIT in HSP remains underexplored.

This case series involved five stroke patients diagnosed with HSP during post-stroke inpatient rehabilitation. Their pain was not responding to analgesics and physical modalities, causing poor rehabilitation participation. All patients received the same PIT regime, whereby 1-5 ml of buffered 5% dextrose was administered subcutaneously to the lateral and intermediate supraclavicular nerve exiting point, quadrangular space, and triangular space. Pain levels, shoulder passive range of motion (PROM), and Fugl-Meyer Assessment for upper extremity (FMA-UE) scores were measured pre- and post-treatment. Standard rehabilitation care continued post-treatment.

All patients reported significant pain reduction, with numerical rating scale (NRS) scores decreasing from an average of 7.3 to 1.0. Shoulder PROM improved by an average of 40 degrees in flexion and 37 degrees in abduction. FMA-UE scores increased from an average of 32.0 to 57.3, with all patients achieving the minimal clinically important difference of 12.4. No adverse effects were reported.

In conclusion, a single-session PIT demonstrated effectiveness in reducing pain and improving function in patients with HSP, facilitating engagement in rehabilitation. This approach may be particularly valuable in facilities lacking ultrasound equipment.

## Introduction

Hemiplegic shoulder pain (HSP) is prevalent among stroke patients, with reported rates ranging from 22 to 47% [1]. It often develops within three months post-stroke and can persist for up to two years [1]. HSP not only hinders upper limb rehabilitation but also diminishes overall function and quality of life [2,3].

Current treatment options for HSP include intra-articular steroid injections, nerve blocks, botulinum toxin injections, hyaluronic acid injections, and prolotherapy [4,5]. However, limited access to credentialed practitioners and ultrasound machines can restrict these treatments, particularly in certain healthcare settings.

Perineural injection therapy (PIT) utilizes dextrose to target subcutaneous nerves that may be responsible for pain [6]. Previous studies have shown promising results for PIT in treating chronic shoulder pain [7], complex regional pain syndrome [8], and adhesive capsulitis [9]. However, the effectiveness of PIT specifically for HSP remains largely unexplored. This study presents our findings on the application of PIT in patients with HSP and highlights its potential as a novel and alternative treatment method.

This study was previously presented as an abstract and won first place at Indonesia Malaysia Regenerative Study Group (IMRSG) Abstract Competition on July 28, 2024.

## Case presentation

This case series included five stroke patients diagnosed with HSP who were admitted to the stroke ward at the stroke rehabilitation ward for post-stroke inpatient rehabilitation in May 2024. This study was approved by the Universiti Malaya Medical Centre Ethics ID: 2022107-11604. Informed consent was obtained from all patients for their participation in the research, and the findings were published in peer-reviewed journals.

Participants

Five patients participated in the study (2 men and 3 women), with an average age of 54 years. All patients reported pain during active movement of the hemiplegic shoulder, worsening beyond a specific range of flexion and abduction (Table 1). 

**Table 1:** Patient characteristics This table summarizes key demographic and clinical characteristics of five patients who experienced strokes and have hemiplegic shoulder pain, participating in this study.

	Patient 1	Patient 2	Patient 3	Patient 4	Patient 5
Age (years)	61	63	60	42	53
Gender	Female	Female	Male	Male	Female
Type of stroke	Left partial anterior circulation infarct	Right lacunar infarct	Right partial anterior circulation infarct	Right lacunar infarct	Right partial anterior circulation infarct
Duration of stroke	3 months	2 months	2 months	3 months	3 months
Site of shoulder pain	Right	Left	Left	Left	Left

The pain experienced by the patients was not neuropathic in nature, and there was no history of injury or trauma to the shoulder. Physical examination revealed limited passive range of motion (PROM) due to pain, reported on a scale of 5/10 to 9/10. Additionally, two patients experienced pain over the dorsal part of the upper arm during passive movement of the upper limb. There was minimal to no spasticity observed. All patients received oral analgesics and physical modalities for one week, but there was minimal to no improvement in pain levels. Consequently, they demonstrated poor participation in upper limb rehabilitation and slow recovery progress. 

Intervention

**Figure 1:**
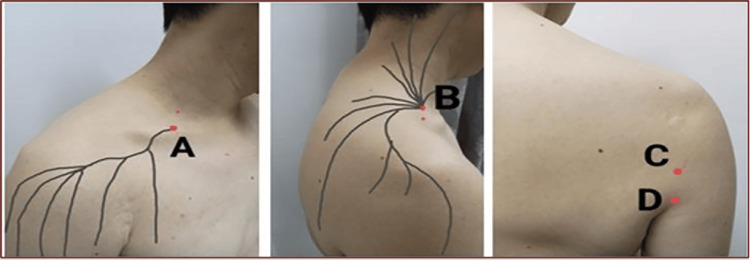
Anatomical Locations for Perineural Injection Therapy The red dot in this figure indicates the site of perineural injection therapy. A: Lateral supraclavicular nerve exit point; B: Intermediate supraclavicular nerve exit point; C: Quadrangular space; D: Triangular space. The locations of points A and B are typically identified through gentle palpation of the subcutaneous tissues above the clavicle in the posterior triangle of the neck. Point A is generally located at the center of the triangle's base, while Point B is found approximately two fingerbreadths above and medial to Point A. A key indicator for locating the chronic constriction injury is a sudden increase in local tenderness, occasionally accompanied by radiation along its distribution, which occurs as the lateral and intermediate branches of the supraclavicular nerve pierce through the investing fascia of the upper trapezius and the platysma fascia.

The PIT protocol for HSP involved palpating cutaneous nerves to identify tender chronic constriction injury (CCI) points before administering dextrose [5]. In order to standardize the protocol, all of these five patients received buffered 5% dextrose without local anesthetics using a 26-gauge 12mm, hypodermic needle (SJ Needle, Malaysia) at the following sites (Figure 1; Video 1).

**Video 1:** Perineural injection technique for hemiplegic shoulder pain This video demonstrates the procedure for perineural injection of 5% dextrose, without local anesthetic, at the four standardized injection points for patients experiencing hemiplegic shoulder pain.

I. 1ml to the lateral supraclavicular nerve exiting point, which supplies the anterior shoulder [10].

ii. 1ml to the intermediate supraclavicular nerve exiting point, which supplies the superior and posterior shoulder [10].

iii. 5mls into quadrangular space, where the axillary nerve passes through supplying the lateral shoulder [10,11].

iv. 5mls into triangular space, where the radial nerve passes through supplying the dorsal part of the upper arm [10,11].

All five patients received the same PIT regime while continuing standard rehabilitation care. Outcomes were assessed through pain scores using the Numerical Rating Scale (NRS), shoulder PROM (measured in degrees for flexion and abduction), and Fugl-Meyer Assessment for upper extremity (FMA-UE) scores at baseline and discharge. The average hospital stay for this group is approximately two weeks, which is also the timeframe for our final assessment.

Outcome measures

Pain was evaluated using the NRS. Shoulder PROM was measured in degrees, and the FMA-UE assessed upper extremity function.

Results

**Figure 2:**
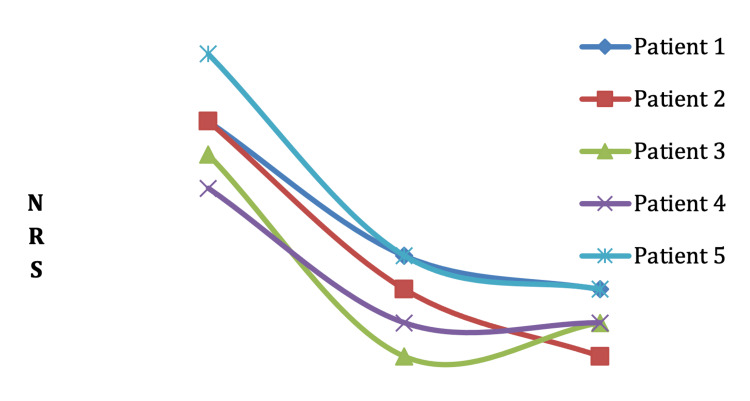
Shoulder pain score using the numeric rating scale (NRS) at pre-PIT, post-PIT, and discharge PIT: Perineural Injection Therapy.

**Figure 3:**
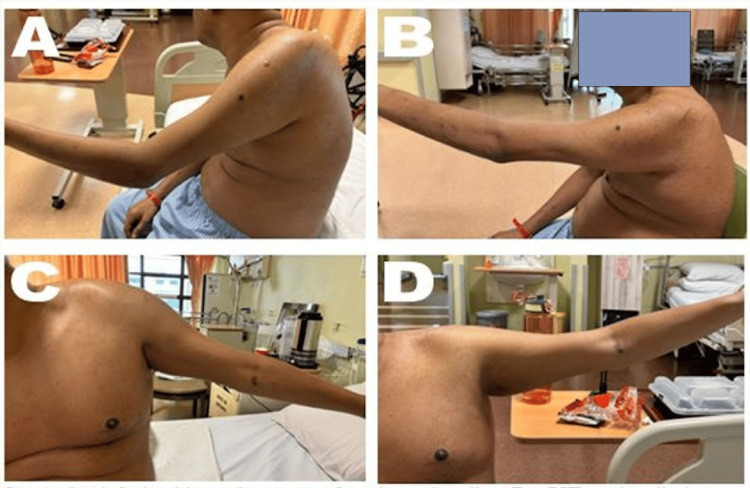
State of the shoulder before and after perineural injection therapy with buffered 5% dextrose without a local anesthetic This figure illustrates the active range of motion of the shoulder at baseline (before perineural injection therapy) and at discharge. A. Shoulder flexion at baseline; B. Shoulder flexion at discharge; C. Shoulder abduction at baseline; D. Shoulder abduction at discharge.

All patients experienced a significant reduction in shoulder pain, with NRS scores decreasing from an average of 7.3 to 1.0 (Figure 2). PROM improved, with average increases of 40 degrees in shoulder flexion and 37 degrees in abduction. FMA-UE scores rose from an average of 32.0 to 57.3, with all patients achieving the minimal clinically important difference of FMA-UE 12.4 [12]. No adverse effects were reported (Figures 2, 3, Table 2). 

**Table 2:** Shoulder passive range of motion (PROM) and Fugl-Meyer Assessment for the upper extremity (FMA-UE) at baseline and discharge This table presents the shoulder passive range of motion (PROM) and the Fugl-Meyer Assessment for the upper extremity (FMA-UE) at two time points: baseline and discharge. The PROM values indicate the maximum range achievable in shoulder movements, while the FMA-UE scores reflect the functional ability and motor performance of the upper extremity. Comparisons between baseline and discharge measurements highlight the changes in patient outcomes following intervention.

Patient	Baseline PROM (Flexion)	Discharge PROM (Flexion)	Baseline PROM (Abduction)	Discharge PROM (Abduction)	Baseline FMA-UE	Discharge FMA-UE
1	120	170	80	130	20	52
2	110	160	90	120	26	56
3	100	150	80	130	48	54
4	120	140	90	120	50	64
5	85	130	90	130	52	68

## Discussion

HSP arises from a complex interplay of neurological and mechanical factors, including paralysis, neuropathic pain, spasticity, shoulder subluxation, muscle imbalance, and soft tissue injuries [2]. Hilton’s law posits that nerves supplying muscles also innervate the joint and overlying skin [13]. Consequently, injuries to cutaneous nerves can lead to deeper structural pain. Muscle imbalance, spasticity, and shoulder subluxation may irritate the nerves, resulting in chronic constrictive injury (CCI). Variations in muscle tone, alongside spasticity, and shoulder strains from subluxation or inappropriate shoulder care can potentially irritate or traumatize the sensory nerves that traverse between muscles and fascial layers. Such sensory nerve injuries often lead to nerve edema both proximally and distally, causing strangulation at points where the nerves penetrate the fascia, ultimately contributing to CCI [14]. Additionally, alterations in fascial tension can exacerbate CCI [6]. This disruption in nerve circulation adversely affects nerve health and repair processes [14]. Furthermore, ischemia and hypoglycemia in nerves can increase C-fiber firing, resulting in neurogenic inflammation [15]. The injection of buffered 5% dextrose into the perineural region specifically targets these C fibers to mitigate neurogenic inflammation. This intervention can decrease nerve edema, relieve CCI constrictions, restore nerve circulation and function, and ultimately provide pain relief [16]. This mechanistic understanding supports the effectiveness of PIT in managing HSP.

Recent trends in ultrasound-guided musculoskeletal injections have improved the precision of such interventions [17,18]. This technique provides real-time visualization of surrounding structures, enhancing needle placement accuracy and reducing the risk of injuring nerves and blood vessels [19]. In Malaysia and many countries, many rehabilitation or musculoskeletal physicians are trained to perform these injections under ultrasound guidance. Various minimally invasive interventions, including nerve block, intra-articular injections, and prolotherapy, are routinely performed for stroke patients with musculoskeletal disorders [5]. However, several challenges associated with these interventions have been identified. 

First, the time to intervention can be prolonged due to several factors. These include the need for diagnostic ultrasound to identify musculoskeletal pathologies, the necessity of withholding antiplatelet or anticoagulant medications for 1-3 days to minimize hematoma risk during injection, and the scheduling of the intervention itself. Both diagnostic evaluations and scheduling heavily on the availability of the trained physicians performing the procedure and the ultrasound facilities. 

Second, stroke patients with musculoskeletal disorders may require extended use of oral analgesics, to facilitate participation in rehabilitation until the intervention can be performed. Unfortunately, options for analgesics are limited for these patients. Nonsteroidal anti-inflammatory drugs pose an increased cardiovascular risk [20], while weak opioids like tramadol can lead to central nervous system side effects, including sedation, headache or seizures, and gastrointestinal side effects, such as nausea, vomiting, or constipation. [21]. 

Third, the needle to withhold antiplatelet or anticoagulant medications may place stroke patients at an increased risk of recurrent strokes [22]. 

In contrast, PIT can be administered immediately, significantly reducing the time to intervention. The five patients in this case series were able to engage in upper limb exercises sooner, without interruption from pain, expediting their rehabilitation progress and shortened their length of stay. This rapid intervention not only enhances the overall efficiency of rehabilitation but also provides a critical alternative for patients in district hospitals or non-hospital-based rehabilitation centers that lack ultrasound facilities. 

While this case series demonstrates the effectiveness of PIT for HSP, it is important to acknowledge its limitations. The authors are sharing our experience with a specific group of patients, those experiencing HSP post-stroke. The results presented here, derived from a limited number of patients, should not be generalized until further research is conducted, including larger-scale randomized controlled trials and extended follow-up periods to assess the sustainability of pain relief and functional improvement post-discharge. Additionally, studies in various regions of the world are essential to establish a standardized protocol for treating HSP in post-stroke patients.

## Conclusions

This case series highlights the effectiveness of a single session of PIT in managing HSP in stroke patients. The intervention resulted in significant pain reduction, enabling patients to engage more actively in upper limb rehabilitation. Notably, improvements were observed in the PROM and FMA-UE scores at the two-week discharge assessment, with minimal to no recurrence of shoulder pain.

While these findings suggest that PIT is a valuable treatment option, particularly in settings lacking access to ultrasound-guided techniques, it is crucial to acknowledge certain limitations. Our study focused on a specific group of patients, those with post-stroke HSP, which restricts the generalizability of the results. Further research is needed, including larger-scale randomized controlled trials and extended follow-up periods, to assess the sustainability of pain relief and functional improvements across diverse patient populations.
